# Diagnostic performance of chest radiography for pediatric tuberculosis across high- and low-burden settings

**DOI:** 10.3389/fped.2025.1704149

**Published:** 2025-12-16

**Authors:** Alicia Hernanz-Lobo, Juan J. Gómez-Valverde, Ángel Lancharro, Ramón Sánchez-Jacob, José Luis Ribó, H. Simon Schaaf, Lara García Delgado, Daniel Capellán-Martín, David Aguilera-Alonso, Daniel Blázquez-Gamero, Antoni Noguera-Julian, Paula Rodríguez-Molino, Laura Minguell, Matilde Bustillo-Alonso, Antoni Soriano-Arandes, David Gomez-Pastrana, Alberto L. García-Basteiro, Orvalho Augusto, María J. Ledesma-Carbayo, Elisa López-Varela, Begoña Santiago-García

**Affiliations:** 1 Pediatric Infectious Diseases Department, Gregorio Marañón University Hospital, Madrid, Spain; 2 Gregorio Marañón Research Health Institute (IiSGM), Madrid, Spain; 3 Centro de Investigación Biomédica en Red de Enfermedades Infecciosas (CIBER INFEC), Carlos III Health Institute, Madrid, Spain; 4 Translational Research Network in Pediatric Infectious Diseases (RITIP), Madrid, Spain; 5 Biomedical Image Technologies, ETSI Telecomunicación, Universidad Politécnica de Madrid, Madrid, Spain; 6 Centro de Investigación Biomédica en Red en Bioingeniería, Biomateriales y Nanomedicina (CIBER BBN), Carlos III Health Institute, Madrid, Spain; 7 Pediatric Radiology Department, Gregorio Marañón University Hospital, Madrid, Spain; 8 George Washington School of Medicine, Washington, DC, United States; 9 Department of Radiology and Medical Imaging, Children’s National Hospital, Washington, DC, United States; 10 Radiology Department, Hospital Sant Joan de Déu, University of Barcelona, Barcelona, Spain; 11 Desmond Tutu TB Centre, Department of Pediatrics and Child Health, Stellenbosch University, Cape Town, South Africa; 12 Pediatric Infectious Diseases Unit, Department of Pediatrics, Hospital Universitario 12 de Octubre, Universidad Complutense, Madrid, Spain; 13 Instituto de Investigación Sanitaria Hospital 12 de Octubre (imas12), Madrid, Spain; 14 Infectious Diseases and Systemic Inflammatory Response in Pediatrics, Infectious Diseases Department, Pediatric Research Institute Sant Joan de Déu, Barcelona, Spain; 15 Department of Surgery and Medico-surgical Specialties, Faculty of Medicine and Health Sciences, Barcelona University, Barcelona, Spain; 16 Centro de Investigación Biomédica en Red de Epidemiología y Salud Pública (CIBERESP), Carlos III Health Institute, Madrid, Spain; 17 Department of Infectious Diseases and Tropical Pediatrics, La Paz Hospital, Madrid, Spain; 18 La Paz Research Institute (IdiPAZ), Madrid, Spain; 19 Department of Pediatrics, University Hospital Arnau de Vilanova, Lleida, Spain; 20 Pediatric Infectious Diseases Unit, Department of Pediatrics, University Hospital Miguel Servet, Zaragoza, Spain; 21 Pediatric Infectious Diseases and Immunodeficiencies Unit, Children’s Hospital, Vall D'Hebron Barcelona Hospital Campus, Barcelona, Spain; 22 Infection and Immunity in Children, Vall d’Hebron Research Institute, Barcelona, Spain; 23 Pediatric Neumology Unit, Pediatrics Department, Hospital Jerez de la Frontera, Cádiz, Spain; 24 Research group UNAIR, Jerez de la Frontera, Cádiz, Spain; 25 Centro de Investigação em Saúde de Manhiça (CISM), Maputo, Moçambique; 26 Barcelona Institute for Global Health (ISGlobal), Hospital Clínic - Universitat de Barcelona, Barcelona, Spain; 27 Department of Global Health, University of Washington, Seattle, WA, United States

**Keywords:** Children, tuberculosis, high-burden, low-burden chest radiography, diagnostic accuracy, interobserver variability clinical predictors

## Abstract

**Background:**

Chest radiography (CXR) is the most widely used imaging tool in pediatric tuberculosis (TB) diagnostic pathways, and remains central in current WHO algorithms. However, its standalone diagnostic accuracy has not been well established in standardized multicenter evaluations. This study aimed to determine the diagnostic performance and interobserver agreement of CXR for pediatric TB across two epidemiologically distinct settings, and to assess the added value of clinical information and lateral projections.

**Methods:**

We evaluated the diagnostic performance of CXR in two pediatric cohorts from distinct TB-burden settings. The high-burden cohort (Mozambique) included 218 children under 3 years (10 confirmed TB, 95 unconfirmed TB, 113 unlikely TB). The low-burden cohort (Spain) included 674 children under 18 years (145 confirmed TB, 237 unconfirmed TB, 95 with TB infection, 101 with community-acquired pneumonia, and 96 healthy controls). Four independent expert readers (three pediatric radiologists and one pediatric infectious disease specialist), each with over 15 years of experience, interpreted CXRs using a standardized digital platform, blinded to clinical data. In a subset of 75 Spanish cases, re-readings incorporated limited clinical information.

**Results:**

Sensitivity for confirmed TB was low in both settings (31.0% in Mozambique, 46.1% in Spain), while specificity was high (94.7% and 96.5%, respectively). In a subset of 75 Spanish cases, adding limited clinical data increased sensitivity from 39.3% to 50.0% (*p* = 0.02) and specificity from 88.1% to 97.4% (*p* < 0.001). Among children with lateral views, sensitivity rose from 39.1% to 53.6% (*p* = 0.01), without significant change in specificity. Interobserver agreement for TB-related findings was only fair (ICC 0.29–0.31).

**Conclusions:**

This multicenter analysis confirms the limited sensitivity but high specificity of CXR for pediatric TB, even when interpreted by expert readers. These findings highlight that CXR alone cannot reliably confirm or exclude disease and should be integrated with clinical and microbiological data. Future diagnostic pathways, including artificial intelligence–assisted CXR interpretation, will likely need multimodal approaches to overcome the intrinsic limitations of imaging alone.

## Introduction

Tuberculosis (TB) remains a leading cause of death from a single infectious agent globally, with 167,000 deaths among children under 15 years reported in 2023. The burden is unevenly distributed: TB incidence ranges from fewer than 10 cases per 100,000 population in low-burden regions to over 400 cases per 100,000 in high-burden settings such as sub-Saharan Africa (1). Diagnosing TB in children presents substantial challenges: clinical and radiological features frequently overlap with other respiratory diseases, and microbiological confirmation is often hindered by the difficulty in obtaining respiratory samples and the limited sensitivity of current tests (2). Moreover, most pediatric TB cases occur in low-resource settings, where undiagnosed disease is a major driver of avoidable mortality (3).

Chest x-ray (CXR) remains a key component of pediatric TB diagnosis and is widely used to assess treatment response and disease severity. Typical radiological findings include mediastinal lymphadenopathy, miliary pattern, and airway compression (4, 5). However, multiple studies have highlighted the limited sensitivity and specificity of CXR (6), with normal imaging reported in up to 15%–20% of children with confirmed TB (7, 8), and substantial variability between readers, especially regarding TB-specific features such as lymphadenopathy (8–13).

Various reports (5, 14–16), including primary research and meta-analyses, have provided key evidence on radiological features of pediatric TB, which informed the development of the most recent WHO guidelines (17). These guidelines emphasize the role of CXR within diagnostic algorithms and for disease severity classification, including criteria to guide treatment decisions and eligibility for shorter treatment regimens. In the current diagnostic landscape, ongoing advances in molecular assays, biomarker discovery, and artificial intelligence–based imaging are reshaping approaches to pediatric TB. Nevertheless, chest radiography remains an essential and irreplaceable component of clinical evaluation—widely accessible, cost-effective, and deeply embedded in diagnostic practice (18).

In this study, we evaluated the diagnostic performance of CXR in two well-characterized pediatric cohorts from high- and low-burden settings, assessing diagnostic accuracy, interobserver agreement, and the incremental value of clinical data and lateral projections.

## Methods

### Study cohorts

We conducted a retrospective multi-centre multi-group diagnostic study including two cohorts: a high-TB-burden cohort from Mozambique (estimated TB incidence, 400 cases per 100,000 patients/year) and a low-TB-burden cohort from Spain (estimated TB incidence, 10 cases per 100,000 patients/year) (1) (Supplementary Material 1).

The high-burden cohort included children under three years of age enrolled in the prospective ITACA study [Manhiça Health Research Centre, CISM, Mozambique, 2011–2012 (19, 20)]. Eligibility included TB-compatible symptoms or contact with a smear-positive pulmonary TB adult. Only cases with pulmonary involvement were included. Patients were classified as confirmed TB, unconfirmed TB, or unlikely TB following standardized criteria (21, 22).

The low-burden cohort included children under 18 years classified into four groups: TB disease, TB infection (TBI), community-acquired pneumonia (CAP), and healthy controls (HC). All pulmonary TB cases were included from the Spanish Pediatric TB Research Network (pTBred) (23, 24). Patients with TB were eligible for analysis if they were ≤18 years of age at TB diagnosis and had confirmed or unconfirmed TB according to clinicians, for whom anti-TB treatment was initiated. Children with incomplete microbiological or CXR data, as well as those with exclusively extrapulmonary TB, were excluded. TBI cases were identified from hospital registries at Hospital Gregorio Marañón (HGM) and Hospital 12 de Octubre (Madrid, Spain). CAP cases were selected from the HERACLES surveillance cohort, specifically from HGM, Hospital 12 de Octubre, and Hospital La Paz, focusing on laboratory-confirmed invasive pneumococcal disease (25). HC were selected exclusively from the HGM pediatric radiology database among children undergoing CXR between March 2016 and September 2018 for non-infectious reasons (e.g., pre-surgical assessments, evaluation of foreign body ingestion, or thoracic trauma/pain), frequency-matched to TB cases by age group and sex. Children presenting with fever or respiratory symptoms were excluded. After excluding nine participants (5 TBI, 4 HC) due to incomplete data, the final low-burden cohort included all TB cases, 95 TBI, 101 CAP, and 96 HC.

### Study definitions

Patients were classified according to international definitions (22). Confirmed TB was defined as clinical and/or radiological signs consistent with pulmonary TB with microbiological confirmation (culture and/or nucleic acid amplification test). Unconfirmed TB was considered when microbiological confirmation was lacking, but the patient met at least two of the following criteria: i) symptoms/signs suggestive of TB, ii) CXR findings consistent with TB, iii) close contact with an infectious TB source patient, or iv) positive response to TB treatment. Unlikely TB applied to children not fulfilling criteria for confirmed or unconfirmed TB. The TB disease group included both confirmed and unconfirmed cases.

TBI was defined as asymptomatic children with a normal CXR and evidence of *M. tuberculosis* infection (positive TST or IGRA). CAP cases had clinical and radiological features of pneumonia with microbiological confirmation of *S. pneumoniae* from blood or pleural fluid (25). Severe TB was defined as previously established (16, 17) and included at least one of the following: positive sputum smear for acid-fast bacilli, extrapulmonary TB (excluding isolated peripheral lymph node TB), and severe CXR findings (multilobar involvement, miliary pattern, airway compression, cavities, or complicated pleural effusion).

### Clinical data collection

Clinical data were collected retrospectively using standardized forms and included demographics (age, sex), TB contact, HIV status (if available), nutritional status, presenting symptoms (fever, cough), TST/IGRA results, microbiological data, and clinical outcomes. Data sources were the ITACA database for the high-burden cohort, pTBred for TB cases in the low-burden cohort, and hospital records for TBI, CAP, and HC groups.

### CXR data collection and assessment

All CXRs were obtained at diagnosis and retrieved from the Picture Archiving and Communication System (PACS) of each participating center in Digital Imaging and Communications in Medicine (DICOM) format to ensure optimal image quality. Images were anonymized and uploaded to a purpose-built, secure remote reading platform specifically developed for this study (26). This platform enabled standardized, blinded reading procedures, providing a structured interface, randomized case presentation, and predefined reporting fields.

In the high-burden cohort, all children underwent both posteroanterior (PA) or anteroposterior (AP) and lateral (LAT) projections. In the low-burden cohort, the availability of LAT projections was based on local clinical protocols at each participating hospital, in line with national recommendations (27), but without a standardized imaging protocol within the pTBred registry.

Expert readers assessed image quality (readable/unreadable), TB suggestiveness (suggestive/not suggestive), and the presence of characteristic TB findings and localization (enlarged lymph nodes, airway compression, hyperinflation, collapsed lung/lobe, alveolar opacity, interstitial opacity, miliary pattern, pleural effusion, cavities, calcified parenchymal lesions). Radiological severity was classified *post hoc* using standardized severity criteria (17).

Four independent readers (three pediatric radiologists and one pediatric TB specialist, all with over 15 years of experience) evaluated the images independently, blinded to clinical data and final diagnosis. In the low-burden cohort, a random subset of 75 CXRs was reassessed after a time gap with access to limited clinical data (age, TB contact, symptoms), while remaining blinded to prior readings and diagnosis. Randomization of image order was maintained during both reading rounds via the platform to minimize recall bias.

### Sample size

All eligible cases were included in both cohorts. In the low-burden cohort, approximately 100 patients per comparison group (TBI, CAP, HC) were selected, with final numbers adjusted after exclusion for incomplete data. The second-reading sub-analysis was limited to a randomly selected sample of 75 low-burden cohort patients, chosen to detect a 20% increase in sensitivity with 70% power at a 95% confidence level. This sub-analysis was not performed in the high-burden cohort owing to the limited size of the cohort.

### Statistical analysis

High- and low-burden cohorts were analyzed separately due to differences in study design, age distribution, and diagnostic work-up. Diagnostic performance of CXR was evaluated in terms of sensitivity, specificity, positive predictive value (PPV), and accuracy, using two reference standards: (a) microbiological confirmation (confirmed TB), and (b) clinical TB case definition (confirmed + unconfirmed TB). Accuracy was defined as the proportion of correctly classified cases (true positives and true negatives) among all participants. Unreadable or missing CXRs were excluded from the corresponding analyses; no imputation was performed.

In the high-burden cohort, diagnostic performance was calculated using children with unlikely TB as the reference (non-TB) group. In the low-burden cohort, all non-TB participants (TBI, CAP, and healthy controls) were used as the denominator. Further subgroup analyses in the low-burden cohort assessed diagnostic performance across age groups (≤3 years, 3–10 years, and ≥11 years) and evaluated the contribution of clinical information to CXR interpretation in a subset of patients.

Inter-reader agreement was assessed using the intraclass correlation coefficient (ICC), chosen for its applicability in multi-rater scenarios with multiple radiological features. ICC values were interpreted as follows: 0.0–0.2 slight agreement, 0.2–0.4 fair, 0.4–0.6 moderate, 0.6–0.8 substantial, and 0.8–1.0 high agreement. All estimates were reported with 95% confidence intervals (CI). Statistical analyses were performed using Python (v3.8.8), with SciPy (v1.10.1) and scikit-learn (v0.24.1) libraries.

### Ethics

The study was approved by the Human Research Ethics Committees of HGUGM (Spain) and the National Bioethics Committee for Health (Mozambique). All study procedures adhered to the Declaration of Helsinki.

## Results

### Study participants and CXR availability

In the high-burden cohort, a total of 218 children were included: 10 with confirmed TB, 95 with unconfirmed TB, and 113 classified as unlikely TB. Among them, 87.1% were malnourished and 18.8% were living with HIV.

In the low-burden cohort, 674 participants were evaluated: 145 with confirmed TB, 237 with unconfirmed TB, 95 with TB infection (TBI), 101 with community-acquired pneumonia (CAP), and 96 healthy controls (HC). Malnutrition was reported in 0.5% of participants, and 0.2% were living with HIV. Clinical and demographic characteristics are summarized in Tables 1, 2.

**Table 1:** Chest x-ray child study participant characteristics, high TB-burden cohort.

Participant characteristics	Confirmed TB (*n* = 10)	Unconfirmed TB (*n* = 95)	Unlikely TB (*n* = 113)
Gender (male)	4 (40.0)	51 (53.7)	67 (59.3)
Age in months (median, IQR)	22.0 [13.2–31.5]	20.0 [13.0–26.5]	22.0 [16.0–28.0]
Fever	4 (40.0)	6 (6.3)	5 (4.4)
Cough	5 (50.0)	17 (17.9)	14 (12.4)
Malnutrition	5 (50.0)	81 (85.3)	104 (92.0)
HIV positive	2 (20.0)	35 (36.8)	4 (3.5)
Known TB contact	2 (20.0)	11 (11.6)	4 (3.5)

All reported values correspond to (*n*, %) unless otherwise specified. TB, tuberculosis; IQR, interquartile range; HIV, human immunodeficiency virus.

**Table 2:** Chest x-ray child study participant characteristics, low TB-burden cohort.

Participant characteristics	Confirmed TB (*n* = 145)	Unconfirmed TB (*n* = 237)	TBI (*n* = 95)	CAP (*n* = 101)	Healthy controls (*n* = 96)
Gender (male)	72 (49.7)	122 (51.5)	38 (40)	42 (41.6)	42 (43.8)
Age in years (median, IQR)	3.5 [1.5–10.1]	5.6 [3.2–10.9]	8.0 [5.0–12.4]	3.2 [1.6–5.3]	7.2 [2.1–11.8]
Fever	77 (53.1)	158 (66.7)	5 (5.3)	88 (87.1)	0 (0)
Cough	77 (53.1)	101 (42.6)	11 (11.6)	68 (67.3)	0 (0)
Malnutrition	1 (0.7)	3 (1.3)	0 (0)	0 (0)	0 (0)
HIV positive	1 (0.7)	1 (0.4)	0 (0)	0 (0)	0 (0)
Known TB contact	100 (69.0)	180 (75.9)	67 (70.5)	NA	NA

All reported values correspond to (*n*, %) unless otherwise specified. TB, tuberculosis; IQR, interquartile range; HIV, human immunodeficiency virus; TBI, TB infection; CAP, community-acquired pneumonia; NA, not available.

In the high-burden cohort, both posteroanterior (PA) and lateral (LAT) CXR projections were available for all patients, and all images were rated as readable by the three independent readers.

In the low-burden cohort, PA projection was available for all participants. LAT projection was available in 41.6% of TB cases, 32.6% of TBI cases, 9.1% of CAP cases, and 4.2% of HC. Overall image quality was acceptable for interpretation, with the exception of five CXRs, which were rated unreadable by one of the readers.

### Performance of CXR for diagnosis of TB

Compared with other study groups, TB cases were significantly more likely to be interpreted as suggestive of TB on CXR in both cohorts: 18.7% vs. 5.3% in the high-burden cohort (OR = 4.1), and 37.9% vs. 11.8% in the low-burden cohort (OR = 4.5) (Tables 3, 4).

**Table 3:** Chest x-ray findings, high TB-burden cohort.

Chest x-ray findings	Confirmed TB (*n* = 10) (30 reads)	Unconfirmed TB (*n* = 95) (285 reads)	All TB (*n* = 105) (315 reads)	Unlikely TB (*n* = 113) (339 reads)	*p* *	OR* (95% CI)
CXR consistent with TB	9 (30.0)	50 (17.5)	59 (18.7)	18 (5.3)	<0.001	4.1 (3.1–5.4)
TB-suggestive findings						
Enlarged lymph nodes	8 (26.7)	30 (10.5)	38 (12.1)	10 (2.9)	<0.001	4.5 (3.2–6.3)
Airway compression	2 (6.7)	6 (2.1)	8 (2.5)	3 (1.0)	0.13	2.9 (1.6–5.4)
Hyperinflation (unilateral)	2 (6.7)	4 (1.4)	6 (1.9)	1 (0.3)	0.06	6.6 (3.1–14.1)
Collapsed lobe	2 (6.7)	11 (3.9)	13 (4.1)	2 (0.6)	0.003	7.2 (4.2–12.4)
Alveolar opacity	21 (70.0)	85 (29.8)	106 (33.7)	19 (5.6)	<0.001	8.4 (6.6–10.8)
Interstitial opacity	3 (10.0)	22 (7.7)	25 (7.9)	11 (3.2)	0.01	2.6 (1.8–3.7))
Miliary pattern	1 (3.3)	5 (1.8)	6 (1.9)	0 (0)	0.012	14.3 (6.3–32.4)
Pleural effusion	0 (0)	15 (5.3)	15 (4.7)	0 (0)	<0.001	35.1 (20.6–60.0)
Cavities	0 (0)	3 (1.1)	3 (0.9)	1 (0.3)	0.36	3.3 (1.2–8.8)
Calcified parenchyma	0 (0)	4 (1.4)	4 (1.2)	2 (0.6)	0.44	2.2 (1.0–4.9)

Reported values correspond to the cumulative number of reads by the three readers, and the corresponding proportion relative to the total. Each CXR was read by 3 experts (Expert 1, 2 and 3), generating 3 reads per participant. CXR, chest x-ray; TB, tuberculosis; OR, odds ratio; CI, confidence interval.

* *p-*values based on two-sided χ^2^ tests and ORs, calculated comparing all TB vs. Unlikely TB cases.

**Table 4:** CXR findings, low-burden cohort.

Chest x-ray findings	TB			Controls			Pairwise comparison between TB and controls			
	Confirmed TB (*n* = 145) (434 reads^a^)	Unconfirmed TB (*n* = 237) (707 reads^b^)	All TB (*n* = 382) (1,141 reads^a^^,^^b^)	TBI (*n* = 95) (285 reads)	CAP (*n* = 101) (303 reads)	Healthy (*n* = 96) (288 reads)	All TB vs. TBI*	All TB vs. CAP*	All TB vs. Healthy*	All TB vs. Not-TB**
CXR consistent with TB	200 (46.1)	233 (33.0)	433 (37.9)	13 (4.6)	81 (26.7)	10 (3.5)	<0.001	<0.001	<0.001	<0.001; 4.5 [3.9–5.1]
TB-suggestive findings										
Enlarged lymph nodes	151 (34.8)	165 (23.3)	316 (27.7)	13 (4.6)	30 (10.0)	8 (2.8)	<0.001	<0.001	<0.001	<0.001; 6.1 [5.3–7.1]
Airway compression	53 (12.2)	33 (4.7)	86 (7.5)	0 (0)	19 (6.3)	0 (0)	<0.001	0.534	<0.001	<0.001; 3.6 [2.9–4.5]
Hyperinflation (unilateral)	8 (1.8)	9 (1.3)	17 (1.4)	3 (1.1)	0 (0)	2 (0.7)	0.780	0.032	0.396	0.05; 2.6 [1.7–4.0]
Collapsed lobe	31 (7.1)	29 (4.1)	60 (5.2)	2 (0.7)	23 (7.6)	0 (0)	<0.001	0.127	<0.001	0.01; 1.8 [1.4–2.3]
Alveolar opacity	243 (56.0)	216 (30.6)	459 (40.2)	4 (1.4)	248 (81.8)	4 (1.4)	<0.001	<0.001	<0.001	<0.001; 1.6 [1.4–1.8]
Interstitial opacity	88 (20.3)	89 (12.6)	177 (15.5)	12 (4.2)	38 (12.5)	12 (4.2)	<0.001	0.205	<0.001	<0.001; 2.4 [2.0–2.8]
Miliary pattern	20 (4.6)	9 (1.3)	29 (2.5)	0 (0)	0 (0)	0 (0)	0.003	0.002	0.002	<0.001; 46.3 [31.8–67.5]
Pleural effusion	33 (7.6)	89 (12.6)	122 (10.6)	1 (0.4)	173 (57.1)	0 (0)	<0.001	<0.001	<0.001	<0.001; 0.4 [0.4–0.5]
Cavities	37 (8.5)	11 (1.6)	48 (4.2)	0 (0)	14 (4.6)	0 (0)	<0.001	0.750	<0.001	0.001; 2.6 [2.0–3.5]
Calcified parenchyma	31 (7.1)	31 (4.4)	62 (5.4)	0 (0)	1 (0.3)	1 (0.3)	<0.001	<0.001	<0.001	<0.001; 25.0 [19.2–32.5]

Reported values correspond to the cumulative number of reads by the three readers. Each CXR was read by 3 experts (Expert 1,2 and 4), generating 3 reads per participant. CXR, chest x-ray; TB, tuberculosis; TBI, tuberculosis infection; CAP, community acquired pneumonia; OR, odds ratio; CI, confidence interval.

a Among Confirmed TB, one CXR was considered unreadable by Expert 4.

b Among Unconfirmed TB, 4 CXRs were considered unreadable by Expert 4.

* *p-*values based on two-sided χ^2^ tests.

** *p-*values based on two-sided χ^2^ tests and ORs, calculated comparing TB vs. Not TB (TBI + CAP + healthy controls).

**Figure 1:**
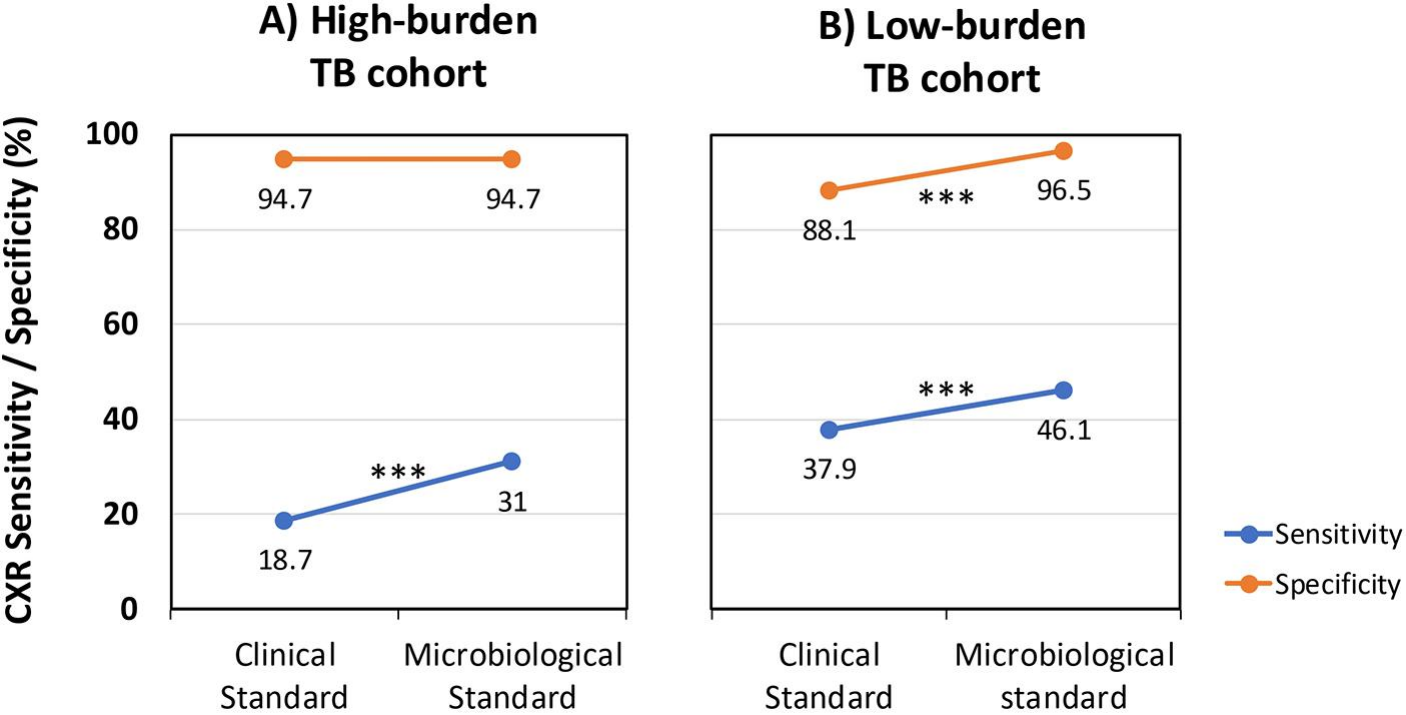
Diagnostic performance of CXR in pediatric TB across high- and low-burden settings. **(A)** Sensitivity and specificity in a high-burden cohort. **(B)** Sensitivity and specificity in a low-burden cohort. Sensitivity (blue) and specificity (orange) are shown for children with clinically defined TB and those with microbiologically confirmed disease. Lines connect paired results within each cohort. TB, tuberculosis; CXR, chest x-ray; ***p < 0.001.

Despite this difference, the overall sensitivity of CXR for TB diagnosis was low in both settings. In the high-burden cohort, sensitivity was 18.7% when using the TB case definition and increased to 31.0% when using microbiological confirmation as the reference (*p* < 0.001). In the low-burden cohort, sensitivity improved from 37.9% to 46.1% when using the microbiological standard (*p* < 0.001) (Table 5, Figure 1).

**Table 5:** Diagnostic performance of expert readers for TB diagnosis, high and low-burden cohort.

Diagnostic performance	Reader	High-burden cohort			Low-burden cohort		
		Microbiological standard (Prevalence 8.1%)	Clinical standard (Prevalence 50.4%)	*p*	Microbiological standard (Prevalence 60.1%)	Clinical standard (Prevalence 79.9%)	*p*
Sensitivity (95% CI)	All	31.0 (17.3–49.2)	18.7 (15.0–23.6)	<0.001	46.1 (41.5–50.8)	37.9 (35.2–40.8)	<0.001
	Reader 1	30.0 (10.8–60.3)	16.4 (10.5–24.6)	0.005	68.3 (60.3–75.3)	57.6 (52.6–62.4)	0.003
	Reader 2	20.0 (5.7–51.0)	12.4 (7.4–20.0)	0.072	34.5 (27.2–42.5)	25.9 (21.8–30.5)	0.014
	Reader 3	44.44 (18.9–73.3)	28.2 (20.4–37.5)	0.003	–	–	–
	Reader 4	–	–	–	35.4 (28.18–43.5)	30.2 (25.8–35.1)	0.146
Specificity (95% CI)	All	94.7 (91.7–96.6)	94.7 (91.7–96.6)	1.0	96.5 (93.7–98.1)	88.1 (85.8–90.1)	<0.001
	Reader 1	94.6 (88.8–97.5)	94.6 (88.8–97.5)	1.0	99.0 (94.3–99.8)	78.4 (73.4–82.8)	<0.001
	Reader 2	98.2 (93.8–99.5)	98.2 (93.8–99.5)	1.0	99.0 (94.3–99.8)	92.8 (89.3–95.2)	<0.001
	Reader 3	91.1 (84.3–95.1)	91.1 (84.3–95.1)	1.0	–	–	–
	Reader 4	–	–	–	91.7 (84.4–95.7)	93.1 (89.6–95.5)	0.488
PPV (95% CI)	All	33.3 (18.6–52.2)	76.6 (66.0–84.7)	<0.001	95.2 (91.5–97.4)	80.6 (77.1–83.8)	<0.001
	Reader 1	33.3 (12.1–64.6)	73.9 (53.5–87.5)	<0.001	99.0 (94.6–99.8)	77.7 (72.5–82.2)	<0.001
	Reader 2	50.0 (15.0–85.0)	86.7 (62.1–96.3)	<0.001	98.0 (89.7–99.7)	82.5 (74.7–88.3)	<0.001
	Reader 3	28.6 (11.7–54.6)	74.4 (58.9–85.4)	<0.001	–	–	–
	Reader 4	–	–	–	86.4 (75.5–93.0)	85.1 (78.1–90.1)	0.600
Accuracy (95% CI)	All	89.6 (86.1–92.3)	58.2 (54.4–62.0)	<0.001	66.2 (62.7–69.6)	59.7 (57.5–61.8)	0.002
	Reader 1	89.3 (82.6–93.7)	56.9 (50.3–63.4)	<0.001	80.5 (75.0–85.0)	66.6 (63.0–70.1)	<0.001
	Reader 2	91.9 (85.7–95.5)	56.9 (50.2–63.3)	<0.001	60.2 (53.9–66.1)	54.9 (51.1–58.6)	0.153
	Reader 3	87.6 (80.6–92.3)	60.9 (54.3–67.2)	<0.001	–	–	–
	Reader 4	–	–	–	57.9 (51.6–64.0)	57.5 (53.7–61.2)	0.912

Diagnostic performance was tested against a microbiological standard (confirmed TB) and a clinical standard (confirmed TB + unconfirmed TB). TB, tuberculosis; CI, confidence interval; PPV, positive predictive value.

Specificity remained high in both cohorts regardless of the reference standard, with values of 94.7% in the high-burden cohort and 96.5% in the low-burden cohort (*p* > 0.05). The positive predictive value (PPV) was substantially higher in the low-burden cohort (95.2%) than in the high-burden cohort (33.3%), reflecting the differences in pre-test probability.

Subgroup analysis by age was performed in the low-burden cohort. Sensitivity remained consistent across age groups: 36.7% in children <3 years, 36.2% in those aged 3–11 years, and 42.7% in those ≥11 years. Specificity also remained stable, with values of 88.0%, 86.8%, and 90.4%, respectively. No statistically significant differences were observed across age groups.

### CXR findings across diagnostic categories

Children with TB showed a higher frequency of radiological features typically associated with the disease (Tables 3, 4). The most common abnormality in both cohorts was alveolar opacity (33.7% in the high-burden cohort and 40.2% in the low-burden cohort), followed by lymphadenopathy (12.1% and 27.7%, respectively) and interstitial opacity (7.9% and 15.5%, respectively).

In the high-burden cohort, TB cases were significantly more likely than unlikely TB cases to present with pleural effusion (OR 35.1; 95% CI 20.6–60.0), miliary pattern (OR 14.3; 95% CI 6.3–32.4), alveolar opacities (OR 8.4; 95% CI 6.6–10.8), and lung or lobe collapse (OR 7.2; 95% CI 4.2–12.4).

In the low-burden cohort, TB cases also showed higher odds of TB-related findings compared to non-TB cases, particularly calcified lesions (OR 25.0; 95% CI 19.2–32.5), lymphadenopathy (OR 6.1; 95% CI 5.3–7.1), and airway compression (OR 3.6; 95% CI 2.9–4.5).

A direct comparison between TB and CAP cases in the low-burden cohort showed that alveolar opacities (40.2% vs. 81.8%; *p* < 0.001) and pleural effusion (10.6% vs. 57.1%; *p* < 0.001) were significantly more frequent in CAP than in TB, highlighting some discriminative potential between both conditions.

### Added diagnostic value of clinical context and lateral view in CXR interpretation

**Figure 2:**
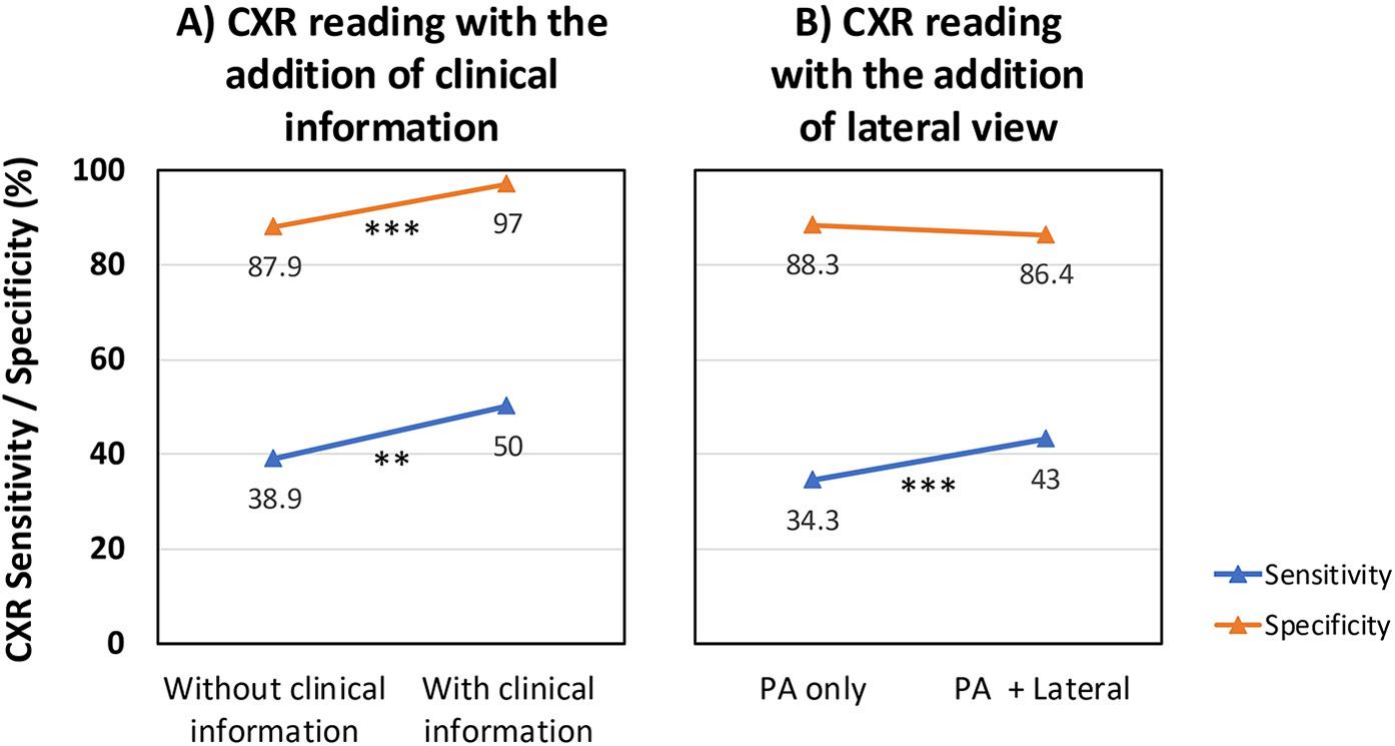
Effect of adding clinical information or a CXR lateral view on the diagnostic performance of CXR. **(A)** Sensitivity and specificity for CXR readings with and without clinical information (low-burden cohort, *n* = 75). **(B)** Sensitivity and specificity for CXR readings with and without a lateral projection (both cohorts, ∼200 children with available projections). Sensitivity (blue) and specificity (orange) are displayed for each reading condition. CXR, chest x-ray; PA, posteroanterior view; **p = 0.02; ***p < 0.001.

In a subset of patients from the low-burden cohort, a second CXR reading was performed after providing expert readers with limited clinical information. This addition improved overall diagnostic performance for TB: sensitivity increased from 38.9% to 50.0% (*p* = 0.02), specificity improved from 87.9% to 97.0% (*p* < 0.001), and overall accuracy rose from 60.4% to 70.7% (*p* = 0.02) (Table 6, Figure 2).

**Table 6:** Diagnostic accuracy of expert readers for TB diagnosis with additional clinical information, low-burden cohort.

Diagnostic performance	Reader	Without clinical information	With clinical information	*p*
Sensitivity (95% CI)	All	38.9 (30.8–47.6)	50.0 (41.4–58.6)	0.02
	Reader 1	57.1 (42.2–70.9)	50.0 (35.5–64.5)	0.38
	Reader 2	33.3 (21.0–48.5)	57.1 (42.2–70.9)	0.003
	Reader 4	26.2 (15.3–41.1)	42.8 (29.1–57.8)	0.03
Specificity (95% CI)	All	87.9 (80.0–92.9)	97.0 (91.5–99.0)	<0.001
	Reader 1	75.76 (59.0–87.2)	93.9 (80.4–98.3)	0.001
	Reader 2	93.9 (80.4–98.3)	96.0 (84.7–99.5)	0.37
	Reader 4	93.9 (80.4–88.3)	100 (89.6–100.0)	0.03
Accuracy (95% CI)	All	60.4 (53.9–66.6)	70.7 (64.4–76.2)	0.02
	Reader 1	65.1 (54.1–75.1)	69.3 (58.2–78.6)	0.60
	Reader 2	60.0 (48.7–70.3)	74.7 (63.8–83.1)	0.05
	Reader 4	56.0 (44.8–66.7)	68.0 (56.8–77.5)	0.13

Reported values corresponding to the diagnostic performance change in a second read incorporating clinical information in a group of 75 children from the low-burden cohort, using clinical diagnosis as reference standard. TB, tuberculosis; CI, confidence interval.

The inclusion of LAT projections also influenced CXR interpretation. Among patients with both PA and LAT views available, the detection of lymphadenopathy increased significantly (14.3% with PA alone vs. 27.3% with PA + LAT; *p* < 0.001), and readers more frequently classified images as suggestive of TB (22.4% vs. 36.6%; *p* < 0.001). Sensitivity improved from 34.3% (PA alone) to 43.0% (PA + LAT; *p* < 0.001), while specificity remained stable (88.3% vs. 86.4%; *p* = 0.24). Notably, accuracy decreased from 62.7% to 52.5% (*p* < 0.001) (Figure 2), likely due to increased false positives among non-TB cases with minor or ambiguous findings on the lateral view.

### Interobserver agreement

The interobserver agreement for classifying a CXR as suggestive of TB was fair in both cohorts, with an ICC of 0.29 in the high-burden cohort and 0.31 in the low-burden cohort (Table 7). Agreement was substantial for the identification of alveolar opacities (ICC = 0.62 in the high-burden and 0.65 in the low-burden cohort) and pleural effusion (ICC = 0.59 and 0.80, respectively). However, agreement for most other radiological findings ranged from slight to fair.

**Table 7:** Inter-reader agreement for TB suspicion, TB-associated findings, and disease severity.

Chest x-ray findings	High-burden cohort (*n* = 218)	Low-burden cohort (*n* = 674)
Consistent with TB	0.29 (0.22–0.39)	0.31 (0.26–0.35)
TB-suggestive findings		
Enlarged lymph nodes	0.20 (0.13–0.30)	0.27 (0.22–0.32)
Airway compression^a^	0.00 (−0.08–0.07)	0.22 (0.17–0.27)
Hyperinflation^a^	0.14 (0.06–0.23)	0.04 (−0.01–0.08)
Collapsed lobe^a^	0.19 (0.10–0.28)	0.09 (0.05–0.14)
Alveolar opacity	0.62 (0.55–0.68)	0.65 (0.61–0.68)
Interstitial opacity	0.00 (−0.08–0.08)	0.11 (0.07–0.16)
Miliary pattern^a^	0.16 (0.08–0.25)	0.37 (0.33–0.42)
Uncomplicated pleural effusion	0.59 (0.52–0.66)	0.80 (0.77–0.82)
Complicated pleural effusion^a^	0.36 (0.27–0.44)	0.60 (0.56–0.64)
Cavities^a^	0.00 (−0.07–0.08)	0.53 (0.49–0.57)
Calcified parenchyma	0.00 (−0.07–0.08)	0.14 (0.09–0.18)

Reported values correspond to intra-class correlation coefficient with 95% confidence interval. TB, tuberculosis.

a Radiological features associated with severe disease.

In the high-burden cohort, the evaluation of severity-related findings showed limited consistency, with slight to fair agreement for airway compression, unilateral hyperinflation, lung or lobe collapse, miliary TB, and cavities. In the low-burden cohort, agreement remained poor across most severity variables, except for complicated pleural effusion (moderate, ICC = 0.60) and cavities (moderate, ICC = 0.53).

## Discussion

In this large study including two well-characterized pediatric cohorts from high- and low-burden TB settings, we observed consistently low sensitivity and high specificity of chest radiography for the diagnosis of pediatric TB. Even when interpreted by expert readers, agreement was only fair for TB-specific features, underlining the intrinsic limitations of CXR as a stand-alone diagnostic tool. Notably, sensitivity increased when focusing on microbiologically confirmed TB cases and when clinical information or a lateral projection was provided, suggesting that contextual data remain essential for improving radiological performance.

The frequency of radiographic abnormalities was higher in children with confirmed TB across both cohorts. As previously reported (28), alveolar opacification was the most frequent abnormality, followed by enlarged lymph nodes and interstitial opacification. Differences between cohorts likely reflected the younger age and higher prevalence of HIV and malnutrition in the Mozambican cohort, in which cavities and pleural effusions were infrequent, in contrast to older children in the Spanish cohort. In addition, the lower sensitivity observed in the high-burden setting may partly reflect differences in inclusion criteria: in Mozambique, all children with clinical suspicion or TB contact were enrolled regardless of disease severity or radiological findings, leading potentially to the inclusion of many mild or subclinical cases with subtle imaging changes. Frequent comorbidities and technical limitations may have further obscured classical TB features. These findings reinforce the need to interpret radiographic patterns within the clinical and epidemiological background of each population.

Comparison with children presenting with community-acquired pneumonia in the low-burden cohort further highlighted diagnostic overlap. Enlarged lymph nodes and calcifications were more common in TB, whereas alveolar opacification and pleural effusion predominated in CAP. This overlap illustrates the difficulty of relying on CXR to distinguish TB from other respiratory diseases, particularly in children, and underscores the importance of combined diagnostic algorithms.

The limited sensitivity of CXR observed in our study—below 50% in both settings—is consistent with prior evidence (5). Previous reports in high-burden regions showed slightly higher sensitivities, including that of Berteloot et al. (11), who reported sensitivity of 71.4% in a multinational study in children living with HIV, or Kaguthi et al. (10), who reported sensitivities ranging from 50% to 75% in infants in Kenya. his limitation poses a risk of underdiagnosis if CXR is used as the main diagnostic modality. By contrast, specificity remained consistently high, indicating that normal CXR findings in children without TB are generally reliable. Our results also demonstrate that the addition of lateral projections and limited clinical information substantially improved sensitivity and accuracy, in line with WHO recommendations advocating for integrated, algorithm-based approaches (14).

Our study revealed limited consensus among highly skilled readers for interpreting TB-associated findings and establishing a diagnosis of TB. The highest agreement was observed for alveolar opacification (high-burden ICC = 0.62, low-burden ICC = 0.65) and pleural effusion (high-burden ICC = 0.59; low-burden ICC = 0.80), both of which are associated with other respiratory infections, such as CAP. Poor agreement was observed for other TB-specific findings, including enlarged lymph nodes (high-burden ICC = 0.20, low-burden ICC = 0.27), miliary TB (high-burden ICC = 0.16; low-burden ICC = 0.37), and those defining disease severity.

Interobserver agreement in our cohorts was modest overall, with substantial variability across TB-related features. In previous studies, interobserver agreement varies widely but is often poor, ranging from 0.15 to 0.39–13. However, Palmer et al. (7) identified moderate agreement (kappa >0.4) for most CXR features, likely owing to their readers' extensive experience working together. Lozano-Acosta et al. (29) reported an overall kappa agreement of 0.51, although their study had a low percentage of CXRs consistent with TB and lacked comparison with gold standard tests. Additionally, Cleveland et al. (30) investigated HIV-exposed or HIV-positive children and reported moderate to high agreement for specific CXR findings. The use of ICC, which capture multi-rater variability more robustly than pairwise kappa, may partly explain the lower agreement estimates compared to earlier reports, but also strengthens the reliability of our conclusions.

The diagnostic yield of CXR can be enhanced through integration with complementary imaging modalities. Computed tomography (CT) provides superior anatomical detail for radiographic–clinical correlation, though access remains limited in many settings. The growing use of Point-of-care ultrasound (POCUS) in pediatric infectious diseases offers a practical adjunct for detecting pleural or parenchymal involvement, expanding the interpretive context and complementing CXR-based evaluation. Beyond conventional interpretation, artificial intelligence (AI)–based tools are emerging as promising adjuncts for pediatric TB diagnosis (31, 32). Early studies suggest that pediatric-specific models could improve sensitivity while maintaining high specificity, particularly for features such as mediastinal lymphadenopathy (33). They may also expand access in high-burden, resource-limited settings (34). However, our study shows that even under ideal conditions, CXR sensitivity remains markedly limited, suggesting that AI relying solely on imaging will likely face an intrinsic ceiling of diagnostic performance. Integrating AI with clinical, epidemiological, and microbiological data is therefore essential for meaningful improvements in pediatric TB detection.

Although our analysis focused on Mozambique and Spain, the findings are likely applicable to other low- and middle-income settings. The diagnostic limitations and reader variability observed in our study reflect challenges common to many high-burden pediatric TB contexts, supporting the broader relevance of these results for strengthening diagnostic algorithms and radiological training in LMIC healthcare systems.

Our study presents notable strengths, mainly that it compares the performance of CXR in high and low-burden cohorts and is one of the largest to assess pediatric TB through CXR. We utilized a standardized and remote platform to assess CXR, potentially enhancing accuracy, and conducted a second read incorporating clinical information. The inclusion of HCs and children with microbiologically confirmed CAP adds depth to our comprehensive performance evaluation. Despite these strengths, our study is subject to limitations, including varied original cohort designs, inclusion criteria, and age compositions, all of which hinder direct cohort-to-cohort comparisons. The distinct designs and inclusion criteria of the two cohorts inevitably introduced heterogeneity in age distribution and disease presentation. These differences may have influenced apparent diagnostic performance, particularly sensitivity. To minimize bias, analyses were conducted separately for each cohort, using standardized blinded readings and harmonized data definitions across sites. The retrospective nature of image selection and the limited sample size in the high-burden cohort may constrain the precision of sensitivity estimates. The use of different third radiologists for each cohort introduces potential variability, and the choice of ICC over kappa may impact comparisons with other studies, even though it enhances data quality and robustness. Future research should aim to include larger, prospectively enrolled pediatric cohorts across diverse endemic settings, apply harmonized reading protocols, and explore the integration of AI-assisted and multimodal imaging approaches to improve diagnostic accuracy and reproducibility.

## Conclusion

In the current landscape, with WHO guidelines placing CXR at the forefront for evaluating disease severity and initiating short-course regimens in pediatric TB, our study underscores the limitations of CXR as a standalone diagnostic tool, even when interpreted by expert readers. Nonetheless, its consistently high specificity highlights its value for ruling out disease across settings with different TB burdens. Importantly, sensitivity improved when incorporating clinical information or lateral projections, reinforcing the need for integrated diagnostic approaches. Taken together, these findings emphasize that optimal strategies for pediatric TB diagnosis should combine radiological assessment with clinical, microbiological, and, potentially, AI–based tools to overcome the intrinsic limitations of imaging alone and improve patient outcomes.

## Data Availability

The raw data supporting the conclusions of this article will be made available by the authors, without undue reservation.
