# Supplementary material for: Diagnostic performance of chest radiography for pediatric tuberculosis across high- and low-burden settings
Source: Front Pediatr. 2025 Dec 16;13:1704149. doi: 10.3389/fped.2025.1704149 (PMC12748184; doi:10.3389/fped.2025.1704149)
Supplement: Supplementary file 1 [file Table1.docx]

# Supplementary Table S1. Inclusion and Exclusion Criteria by Cohort

| **Cohort** | **Group** | **Inclusion criteria** | **Exclusion criteria** | **Setting** |
| --- | --- | --- | --- | --- |
| **Mozambique**  **(high-burden)** | **TB suspected cases** | Children <3 years enrolled in the ITACA study^19-20^; clinical or radiological signs suggestive of TB, or close TB contact. | Exclusively extrapulmonary TB; incomplete data. | Manhica (CISM) |
| **Spain**  **(low-burden)** | **TB disease** | Children and adolescents ≤18 years enrolled in pTBred database^23,24^; confirmed or unconfirmed TB according to clinicians; anti-TB treatment initiated. | Exclusively extrapulmonary TB; unavailable CXR, incomplete microbiological data. | pTBred |
|  | **TB infection** | Asymptomatic children and adolescents ≤18 years; normal CXR; positive TST or IGRA. | Clinical symptoms; abnormal CXR; incomplete data. | Madrid (HGM, H12O) |
|  | **Community-acquired pneumonia** | Children and adolescents ≤18 years with laboratory-confirmed pneumococcal pneumonia enrolled in HERACLES cohort^25^ (blood or pleural fluid). | No microbiological confirmation. | Madrid (HGM, H12O, HULP) |
|  | **Healthy controls** | Children and adolescents ≤18 years undergoing CXR for non-infectious reasons: pre-surgical assessment, foreign body, trauma/pain. | Fever or respiratory symptoms; chronic disease; artifact from medical devices. | Madrid (HGM) |
| TB = tuberculosis; CXR = chest X-ray; TST = Tuberculin Skin Test; IGRA = interferon-gamma release assay; CISM = Centro de Investigação em Saúde de Manhiça HGM = Hospital Universitario Gregorio Marañón; H12O = Hospital Universitario 12 de Octubre; HULP = Hospital Universitario La Paz. | | | | |
